# Clustering CITE-seq data with a canonical correlation-based deep learning method

**DOI:** 10.3389/fgene.2022.977968

**Published:** 2022-08-22

**Authors:** Musu Yuan, Liang Chen, Minghua Deng

**Affiliations:** ^1^ Center for Quantitative Biology, Academy for Advanced Interdisciplinary Studies, Peking University, Beijing, China; ^2^ Department of Probability and Statistics, School of Mathematical Sciences, Peking University, Beijing, China; ^3^ Center for Statistical Science, Peking University, Beijing, China

**Keywords:** bioinformatics, genomics, transcriptomics, omics, statistical, data integration, artificial intelligence, genetics

## Abstract

Single-cell multiomics sequencing techniques have rapidly developed in the past few years. Among these techniques, single-cell cellular indexing of transcriptomes and epitopes (CITE-seq) allows simultaneous quantification of gene expression and surface proteins. Clustering CITE-seq data have the great potential of providing us with a more comprehensive and in-depth view of cell states and interactions. However, CITE-seq data inherit the properties of scRNA-seq data, being noisy, large-dimensional, and highly sparse. Moreover, representations of RNA and surface protein are sometimes with low correlation and contribute divergently to the clustering object. To overcome these obstacles and find a combined representation well suited for clustering, we proposed scCTClust for multiomics data, especially CITE-seq data, and clustering analysis. Two omics-specific neural networks are introduced to extract cluster information from omics data. A deep canonical correlation method is adopted to find the maximumly correlated representations of two omics. A novel decentralized clustering method is utilized over the linear combination of latent representations of two omics. The fusion weights which can account for contributions of omics to clustering are adaptively updated during training. Extensive experiments over both simulated and real CITE-seq data sets demonstrated the power of scCTClust. We also applied scCTClust on transcriptome–epigenome data to illustrate its potential for generalizing.

## 1 Introduction

High-throughput single-cell sequencing technology represented by single-cell RNA sequencing has been widely used in cancer tumors and embryonic development in recent years. The gene expression profile obtained by transcriptome sequencing allows us to study tissue heterogeneity at the individual cellular level. Although the amount of sequencing data is increasing, single-cell RNA sequencing data still meet the characteristics of high noise and dimensions, which limits the accuracy of the calculation method to a certain extent. The recent rise of single-cell multiomics sequencing technology has allowed researchers to obtain information on the epigenomics, genetics, and proteomics of individual cells at the same time. Representative methods include single-cell cellular indexing of transcriptomes and epitopes (CITE-seq) (Stoeckius et al., (2017), single-cell DNA methylation and transcriptome (scM&T-seq) (Angermueller et al., (2016), single-nucleus chromatin accessibility and mRNA expression sequencing (SNARE-seq) (Chen et al., (2019), and single-cell gDNA-mRNA sequencing (DR-seq) (Dey et al., (2015). Among the various single-cell multiomics sequencing technologies, CITE-seq allows simultaneous quantification of gene expression and surface proteins by using single-cell RNA sequencing (scRNA-seq) and antibody-derived tags (ADTs) in single cells. CITE-seq data have the great potential of providing us with a more comprehensive and in-depth view of cell states and interactions.

In scRNA-seq data analysis, a crucial step involves clustering cells into subpopulations to facilitate subsequent downstream analysis. Emerging multiomics CITE-seq data can potentially enrich cell type-specific information across different omics, yet clustering methods still need to be tailored to fully utilize these abundant, yet complex, data sets. Although surface protein expression profiles hold weaker sparsity and lower dimensionality, which makes it easier for researchers to analyze, CITE-seq data inherit the problems existing in scRNA-seq data such as large dimensions, high sparsity, and high noise. Other difficulties also arose when clustering CITE-seq data. The first involves large differences in dimension between transcriptomics and proteomics data, making it difficult to extract omics-invariant data, which benefits clustering in the same manner from two omics. Second, scRNA-seq and surface protein expression data do not necessarily have a high correlation and are not necessarily equally important to the clustering objective. The most popular way of handling multiomics data is to linearly combine omics representations. However, roughly summing representations of low correlation is very likely to blur the boundaries of clusters and defaults that the contributions of both omics to the clustering object are equivalent.

Recently, several computational methods able to cluster CITE-seq data have been developed. Seurat V4 (Hao et al., 2021) maps the input of scRNA-seq and surface protein expression into a shared low-dimensional space with principal component analysis (PCA) and linearly combines those representations. A weighted nearest neighbors (WNN) graph is constructed on that linear fusion. This forms the basis for the application of the Leiden algorithm to present a clustering result. Seurat V4 received great popularity among biological researchers accounting for its convenience and scalability. However, Seurat separates the dimension reduction, WNN graph construction, and clustering procedures so that the low-dimensional representations of RNA and protein data may not suit for constructing a WNN graph or clustering. Also, the WNN graph will be of poor significance when RNA and protein representations are of low correlation. Moreover, the fusion weights of two representations need to be set manually, yet there is no way to assess the contributions of two omics to the clustering object.

On the other hand, deep generative models have been introduced into single-cell multiomics data clustering analysis and have quickly gained popularity. TotalVI (Gayoso et al., 2021) utilizes a variational autoencoder (VAE) (Kingma and Welling, 2014) to handle gene expression and protein data. A latent variable is introduced to represent the shared information of different modalities, or “cell state”, and the posterior estimation of both modalities is learned by omics-specific encoders. Clusters are inferred using the Leiden algorithm. TotalVI is widely used for clustering large-scale multiomics data. However, similar to Seurat, it also separates the character extraction process from the clustering step, making it hard to obtain latent representations well suited for clustering. Moreover, TotalVI ignores the relationship between the representations of RNA and protein data and simply averages them. This primitive process waives the chance to find a better space for clustering and attaches equivalent importance to both omics, thus being easily affected by the influence of the less informative omics.

In this study, we propose single-cell CITE-seq Cluster (scCTClust) to conduct clustering for CITE-seq data. First, we introduce the zero-inflated negative binomial (ZINB) model to characterize RNA data and build two omics-specific autoencoders to extract omics-specific information from both transcriptomics and proteomics separately. Second, a deep canonical correlation analysis (DCCA) (Andrew et al., 2013) method is utilized to find the maximumly correlated expressions of both omics. The representations of the two omics are linearly combined to a fused representation on which clustering is conducted. The fusion weight referring to the contribution of each omics to the clustering object is adaptively updating during the training process. Third, a novel Cauchy–Schwartz (C-S) divergence-based clustering (Kampffmeyer et al., 2019) module is adopted to effectively divide the fused representation into subgroups. This novel C-S divergence-based clustering encourages the clusters to be separable and compact and pushes the assigning vectors to be orthogonal and close to simplex in $R^{K}$ , where *K* is the cluster number. Notably, our clustering module is truly decentralized and thus more robust than the popular metrics-based clustering methods which highly influenced the quality of initialization. Extensive real data and simulation experiments have been conducted to demonstrate the effectiveness and robustness of scCTClust clustering CITE-seq data. To demonstrate the potential of scCTClust clustering other single-cell multiomics data, we also applied scCTClust and other state-of-the-art multiomics clustering methods on several SNARE-seq data sets and received satisfactory results.

## 2 Materials and methods

**FIGURE 1:**
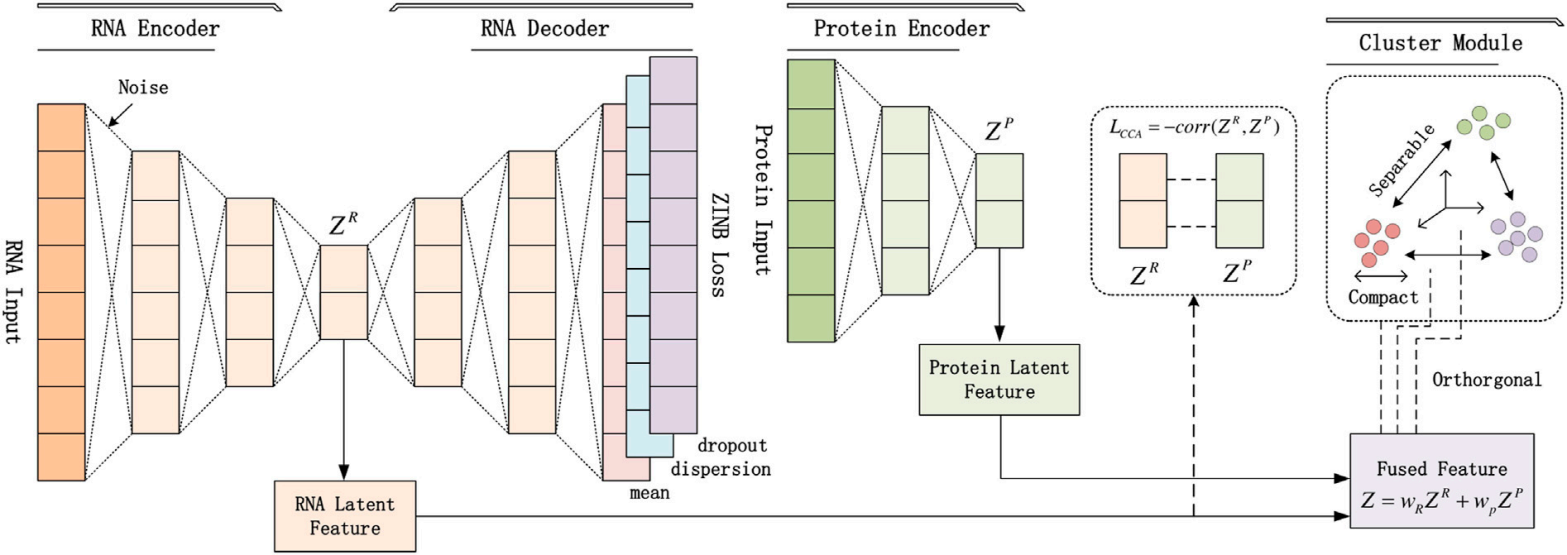
Structure of the scCTClust model: (1) preprocessed multiomics data are used as input of omics-specific encoders separately; outputs are latent features and estimated posterior parameters of the ZINB or NB model. (2) A fusion layer is introduced to linearly fuse the latent features of different omics data. (2) A CCA loss is introduced to find the maximumly correlated omics latent representations. (4) A Cauchy–Schwatz divergence-based clustering module is added after the fusion layer.

The pipeline of our proposed scCTClust is depicted in Figure 1. In the following section, we describe this pipeline in detail.

Suppose our data set consists of *n* samples (cells) observed in transcriptomics and proteomics. Let $x_{i}^{\left(r\right)}$ and $x_{i}^{\left(p\right)}$ be the observation of sample *i* from transcriptomics and proteomics; then the total observation of cell *i* is $\left\{x_{i}^{\left(r\right)},x_{i}^{\left(p\right)}\right\}$ . Given cluster (cell type) number *K*, our objective is to assign the observation set of each cell *i*, $\left\{x_{i}^{\left(r\right)},x_{i}^{\left(p\right)}\right\}$ , to one of the *K* clusters.

### 2.1 Omics-specific autoencoder

First, we transform $x_{i}^{\left(r\right)}$ and $x_{i}^{\left(p\right)}$ to low-dimensional representations $z_{i}^{\left(r\right)}$ and $z_{i}^{\left(p\right)}$ with omics-specific encoder networks *f* ^(*r*)^ and *f* ^(*p*)^ separately.

$$\begin{array}{ll} z_{i}^{\left(r\right)} & =f^{\left(r\right)}\left(x_{i}^{\left(r\right)}\right)\;nonumber\\ z_{i}^{\left(p\right)} & =f^{\left(p\right)}\left(x_{i}^{\left(p\right)}\right). \end{array}$$ (1)

To capture the character of transcriptomic (scRNA-seq) data, we utilize the same ZINB (zero-inflated negative binomial) (Huang et al., 2018) model-based autoencoder as in scSemiCluster (Chen et al., 2021). Protein data are not as sparse as scRNA-seq data; therefore, we empirically use a negative binomial (NB) model, a ZINB model hybrid, to characterize it. Formally, NB is parameterized with a mean (*μ*) and dispersion (*θ*) of the negative binomial distribution, while ZINB is parameterized with an additional coefficient (*π*) that represents the weight of the point mass of probability at zero (the probability of dropout events):

$$\begin{array}{ll} \text{NB}\left(X^{\left(i\right)}|\mu^{\left(i\right)},\theta^{\left(i\right)}\right) & =\frac{\Gamma \left(X^{\left(i\right)}+\theta^{\left(i\right)}\right)}{\Gamma \left(X^{\left(i\right)}+1\right)\Gamma \left(\theta^{\left(i\right)}\right)}\times {\left(\frac{\theta^{\left(i\right)}}{\theta^{\left(i\right)}+\mu^{\left(i\right)}}\right)}^{\theta^{\left(i\right)}}{\left(\frac{\mu^{\left(i\right)}}{\theta^{\left(i\right)}+\mu^{\left(i\right)}}\right)}^{X^{\left(i\right)}}\\ \text{ ZINB }\left(X^{\left(i\right)}|\pi^{\left(i\right)},\mu^{\left(i\right)},\theta^{\left(i\right)}\right) & =\pi^{\left(m\right)}G_{0}\left(X^{\left(i\right)}\right)+\left(1-\pi^{\left(i\right)}\right)NB\left(X^{\left(i\right)}|\mu^{\left(i\right)},\theta^{\left(i\right)}\right), \end{array}$$ (2)

where *X* ^(*i*)^ represents the raw read counts from omics *i*, *i* = *r*, *p*. The omics-specific autoencoder for transcriptomics estimates the parameters *μ* ^(*r*)^, *θ* ^(*r*)^, and *π* ^(*r*)^ by constructing three parallel output layers, and that for protein data estimates *μ* ^(*r*)^, *θ* ^(*r*)^ by constructing two parallel output layers. The loss function of the omics-specific autoencoder is the sum of negative log-likelihoods of according distribution:

$$L_{\text{AE}}=-\log\left(\text{ZINB}\left(X^{\left(r\right)}|\mu^{\left(r\right)},\pi^{\left(r\right)},\theta^{\left(r\right)}\right)\right)-\log\left(\text{NB}\left(X^{\left(p\right)}|\mu^{\left(p\right)},\theta^{\left(p\right)}\right)\right).$$ (3)

We introduce a fusion layer to represent the integrated information of transcriptomics and proteomics, which computes a weight average as given below:

$$z_{i}=w_{r}z_{i}^{\left(r\right)}+w_{p}z_{i}^{\left(p\right)},$$ (4)

where *w*_*r*_, *w*_*p*_ are fusion weights which are contributions of omics to the omic-invariant representation *z*_*i*_. These weights will be adaptively chosen when optimizing the following clustering object.

### 2.2 Canonical correlation analysis

We consider that the latent representations of transcriptomics and proteomics data, *z* ^(*r*)^ and *z* ^(*p*)^, are distributed differently and are often lowly correlated in the latent space. We introduce a canonical correlation analysis (CCA) method to improve the correlation between these two representations and thus get a fusion representation *z* more suited for further clustering.

Let $\left(X_{1},X_{2}\right)\in R^{n_{1}}\times R^{n_{2}}$ denote random vectors with corvariances $\left(\Sigma_{11},\Sigma_{22}\right)$ and cross-corvariance Σ_12_. Typical CCA finds pairs of linear projections of the two views $\left(w_{1}^{\prime }X_{1},w_{2}^{\prime }X_{2}\right)$ that are maximally correlated:

$$\begin{array}{ll} \left(w_{1}^{*},w_{2}^{*}\right) & =\underset{w_{1},w_{2}}{argmax}corr\left(w_{1}^{\prime }X_{1},w_{2}^{\prime }X_{2}\right)\\ & =\underset{w_{1},w_{2}}{argmax}\frac{w_{1}^{\prime }\Sigma_{12}w_{2}}{\sqrt{w_{1}^{\prime }\Sigma_{11}w_{1}w_{2}^{\prime }\Sigma_{22}w_{2}}}. \end{array}$$ (5)

Since the objective is invariant to scaling of *w*_1_ and *w*_2_, the projections are constrained to have unit variance, defined as

$$\left(w_{1}^{*},w_{2}^{*}\right)={argmax}_{w_{1}^{\prime }\Sigma_{11}w_{1}=w_{2}^{\prime }\Sigma_{22}w_{2}=1}w_{1}^{\prime }\Sigma_{12}w_{2}.$$ (6)

When finding multiple pairs of vectors $\left(w_{1}^{i},w_{2}^{i}\right)$ , subsequent projections are also constrained to be uncorrelated with previous ones, that is, $w_{1}^{i}\Sigma_{11}w_{1}^{j}=\;w_{2}^{i}\Sigma_{22}w_{2}^{j}=0$ for *i* < *j*. Assembling the top *k* projection vectors $w_{1}^{i}$ into the columns of a matrix $A_{1}\in R^{n_{1}\times k}$ and similarly placing $w_{2}^{i}$ into $A_{2}\in R^{n_{2}\times k}$ , we obtain the following formulation to identify the top $k\leq \min\left(n_{1},n_{2}\right)\text{ projections: }$

$$\begin{array}{cc} \text{ maximize: } & tr\left(A_{1}^{\prime }\Sigma_{12}A_{2}\right)\\ \text{ subject to: } & A_{1}^{\prime }\Sigma_{11}A_{1}=A_{2}^{\prime }\Sigma_{22}A_{2}=I \end{array}.$$ (7)

There are several ways to express the solution to this objective; we follow the one in the study by Jupp and Mardia (1979). We define $T≜\Sigma_{11}^{-1/2}\Sigma_{12}\Sigma_{22}^{-1/2}$ , and let *U*_*k*_ and *V*_*k*_ be the matrices of the first *k* left- and right-singular vectors of *T*, respectively. Then the optimal objective value is the sum of the top *k* singular values of *T* (the Ky Fan *k*-norm of *T*), and the optimum is attained at

$$\left(A_{1}^{*},A_{2}^{*}\right)=\left(\Sigma_{11}^{-1/2}U_{k},\Sigma_{22}^{-1/2}V_{k}\right).$$ (8)

Back to our scCTClust model, we wish to find proper latent representations of transcriptomics and proteomics that can be maximumly correlated. Instead of finding proper linear projections, we seek appropriate parameters *θ* ^(*r*)^ and *θ* ^(*p*)^ for omics-specific encoders *f* ^(*r*)^ and *f* ^(*p*)^. The according optimization problem is

$$\left(\theta^{\left(r\right)*},\theta^{\left(p\right)*}\right)=\underset{\left(\theta^{\left(r\right)},\theta^{\left(p\right)}\right)}{argmax}corr\left(f^{\left(r\right)}\left(X^{\left(r\right)};\theta^{\left(r\right)}\right),f^{\left(p\right)}\left(X^{\left(p\right)};\theta^{\left(p\right)}\right)\right).$$ (9)

To find $\left(\theta^{\left(r\right)*},\theta^{\left(p\right)*}\right)$ , we follow the gradient of the correlation objective as estimated on the training data. We follow the notation in Eq. 1; let *H*_*r*_ and *H*_*p*_ be the transpose of the latent representations *z* ^(*r*)^ and *z* ^(*p*)^, respectively. It should be noted that the solution in Eq. 8 assumes that the covariance matrices Σ_11_ and Σ_22_ are nonsingular, which is satisfied in practice because they are estimated from data with regularization. We define the centered data matrix ${\bar{H}}_{r}=H_{r}-\frac{1}{n}H_{r},{\bar{H}}_{p}=H_{p}-\frac{1}{n}H_{p}$ and define

$$\begin{array}{cc} {\hat{\Sigma }}_{12} & =\frac{1}{n-1}{\bar{H}}_{r}{\bar{H}}_{p}^{\prime },\\ {\hat{\Sigma }}_{11} & =\frac{1}{n-1}{\bar{H}}_{r}{\bar{H}}_{r}^{\prime }+r_{1}I,\\ {\hat{\Sigma }}_{22} & =\frac{1}{n-1}{\bar{H}}_{p}{\bar{H}}_{p}^{\prime }+r_{2}I \end{array},$$ (10)

where *r*_1_, *r*_2_ > 0 are regularization parameters so that ${\hat{\Sigma }}_{11}$ and ${\hat{\Sigma }}_{22}$ are positive definite.

As is discussed ahead for typical CCA, the total correlation of the top *k* components of *H*_*r*_ and *H*_*p*_ is the sum of the top *k* singular values of the matrix $T={\hat{\Sigma }}_{11}^{-1/2}{\hat{\Sigma }}_{12}{\hat{\Sigma }}_{22}^{-1/2}$ . Using all components, our CCA loss is defined as follows:

$$L_{\mathrm{cca}}=corr\left(H_{r},H_{p}\right)=\|T{\|}_{\text{tr}}=tr{\left(T^{\prime }T\right)}^{1/2}.$$ (11)

### 2.3 C-S divergence-based clustering

To obtain the final cluster assignments, we add a fully connected layer with a softmax activation after the fusion layer to obtain the *k*-dimensional soft label *α*_*a*_.

To perform ideal clustering, we want clusters to be compact and separable, thus making it easy to distinguish among different clusters. We also want cluster assignments, which are K-dimensional soft labels, to be deterministic and close to one-hot vectors. Additionally, the existing metrics-based clustering method, deep embedded clustering (DEC), for an instance, initializes by randomly selecting samples as representatives of clusters and is thus easily affected by bad initialization. Therefore, we wish that the clustering module can be decentralized and robust.

Based on this clustering concept, we designed a deep divergence-based clustering (DDC) loss based on Cauchy–Schwartz divergence (CS divergence) to conduct clustering over *z*_*i*_. DDC loss consists of three parts, ensuring the separability and compactness of clusters, closeness of cluster assignments to simplex corners, and orthogonality of cluster assignments. DDC loss focuses on the relationships between all samples and thereby does not depend on ideal initialization to perform well.

Considering *k* ≥ 2 distinct probability density functions (PDF), CS divergence is defined as

$$D_{\text{cs}}\left(p_{1},\ldots ,p_{k}\right)=-\log\left(\frac{1}{k}\overset{k-1}{\underset{i=1}{\sum }}\underset{j>i}{\sum }\frac{\int p_{i}\left(x\right)p_{j}\left(x\right)dx}{\sqrt{\int p_{i}^{2}\left(x\right)dx\int p_{j}^{2}\left(x\right)dx}}\right).$$ (12)

For a pair of PDFs, *p*_*i*_ and *p*_*j*_, we have $0\leq D_{\text{cs}}\left(p_{1},p_{2}\right)<\infty$ , where we obtain the minimum value if *p*_*i*_ = *p*_*j*_. Assume that we have an *n* × *k* assignment matrix **A** = [*α*_*a*,*i*_]. For the first term of clustering loss, we make use of the divergence in Eq. 12 to measure the distance between clusters. Since the underlying true densities at point *x*, *p*_*i*_(*x*), are unknown, we replace them with soft cluster assignments of cluster *i* for the cell as *a α*_*a*,*i*_ and configure the optimization object with a Gaussian kernel having bandwidth *σ*. To be more explicit,

$$L_{1}=D_{\text{cs}}\left(\alpha_{1},\ldots ,\alpha_{k}\right)=\frac{1}{k}\overset{k-1}{\underset{i=1}{\sum }}\underset{j>i}{\sum }\frac{\alpha_{i}^{T}K\alpha_{j}}{\sqrt{\alpha_{i}^{T}K\alpha_{i}\alpha_{j}^{T}K\alpha_{j}}},$$ (13)

where ***α***_*i*_ is the *i*th column of **A**, the similarity values in **K** follows $\kappa_{ij}=\exp\left(-\|h_{i}-\;h_{j}{\|}^{2}/\left(2\sigma^{2}\right)\right)$ , and *σ* is a hyperparameter. Minimizing $L_{1}$ pushes cosine similarity between cells in the same cluster to be small and the similarity between cells in different clusters to be large, making clusters separable and compact.

For the second term of clustering loss, we make use of the divergence in Eq. 12 to measure the distance between soft cluster assignment vectors for cells and simplex corners. Suppose that ***α***_*a*_ is the *a*th row of **A**, and **e**_*j*_ is corner *j* of the standard simplex in $R^{k}$ . We define an additional term for the loss function *m*_*a*,*j*_ = exp(−‖ ***α***_*a*_ − **e**_*j*_‖) and **m** = [*m*_*a*,*j*_]

$$L_{2}=D_{\text{cs}}\left(m_{1},\ldots ,m_{k}\right)=\frac{1}{k}\overset{k-1}{\underset{i=1}{\sum }}\underset{j>i}{\sum }\frac{m_{i}^{T}Km_{j}}{\sqrt{m_{i}^{T}Km_{i}m_{j}^{T}Km_{j}}},$$ (14)

where **m**_*i*_ is in the *i*th column of **m**. Minimizing this part of loss encourages the cluster assignment vectors to be close to the standard simplex in $R^{k}$ .

The third part is designed as the strictly upper triangular elements of **A** ^*T*^ **A**, where **A** is the *n* × *K* soft assignment matrix,

$$L_{3}=\text{triu}\left(A^{T}A\right).$$ (15)

It consists of inner products between cluster assignment vectors. Cluster assignment vectors are orthogonal if and only if these inner products are zero. Optimizing $L_{3}$ encourages the cluster assignment vectors for different objects to be orthogonal.

The final clustering loss is the sum of these three terms:

$$L_{\text{cluster }}=L_{1}+L_{2}+L_{3}.$$ (16)

Finally, the total loss we use to train scCTClust is

$$L=\gamma L_{\text{AE}}+\delta L_{\text{cca}}+L_{\text{cluster}},$$ (17)

where $L_{\text{cluster }}$ is the clustering loss we defined in Eq. 16, and *γ* and *δ* are hyperparameters which influence the strength of the CCA loss and ZINB loss.

## 3 Results

### 3.1 Simulation studies

ScCTClust is powerful clustering transcriptome–proteome multiomics data. For comparison, we applied scCTClust and several state-of-the-art multiomics clustering methods over a series of simulated CITE-seq data sets generated by R package Splatter. The detailed settings of the simulated datasets are listed in Table 1. Specifically, we aim to investigate the performance of scCTClust against competing methods under different cluster numbers, protein data dimensions, and differential expression feature probabilities. We fix the number of every group of cells at 500 during our experiments. We investigate the performance of each method under different cluster numbers, which varied as 4, 6, 8, and 10, while gene numbers and protein types were fixed at 2500 and 75 differential expression gene/protein probabilities at 0.15 and 0.7. The competing methods include Seurat V4, MOFA+ (Argelaguet et al., 2020), CiteFuse (Kim et al., 2020), BREM-SC, and TotalVI. The performance of each method is evaluated by the commonly used evaluation index in clustering problems ARI and NMI.

**TABLE 1:** Simulation data settings for all experiments; each cluster contains 500 cells; ‘*’ refers to the variable parameter.

Cell/cluster	Cluster	Protein	Prob_RNA_	Prob_protein_
500	*	75	0.15	0.7
500	8	*	0.15	0.7
500	8	75	*	0.7
500	8	75	0.15	*

**FIGURE 2:**
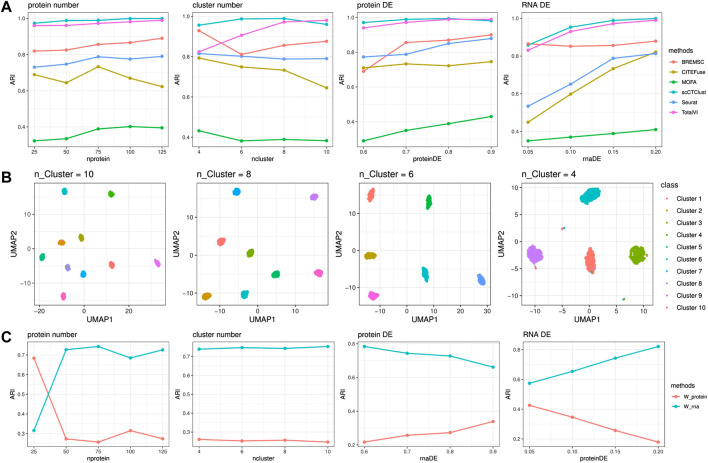
Simulation Experiments. **(A)** Performance of scCTClust and competing methods by ARI over simulated CITE-seq data sets. **(B)** Two-dimensional visualization of latent features extracted by scCTClust using the UMAP dimension reduction method. Only cluster numbers varied in according experiments. **(C)** Behavior of fusion weights during the simulation experiments.

In Figure 2, we can see that the ARI of scCTClust maintained a high level, ranking top 2 of all six methods, across all experiments. When increasing the number of surface proteins, the performance of scCTClust improved, indicating that the neural network-based scCTClust is fully capable of extracting clustering information from omics data of high dimension. While the ARI of Seurat, CiteFuse, and MOFA decreased evidently as the cluster number was larger, the performance of scCTClust maintained an ARI over 0.95. We can also find that when the differential expression probability of a protein is low, scCTClust was not visibly affected as BREM-SC or MOFA was. Similar things happened when the differential expression probability of RNA decreases, which illustrate that scCTClust is less affected by the less informative omics data. To further demonstrate the power of scCTClust, in Figure 2, we plotted the two-dimensional visualization of latent features extracted by scCTClust during the cluster number experiments. The UMAP plot was colored by true labels. scCTClust achieved satisfactory performance, making the clusters compact and separable. We also studied the variation of fusion weights across the experiments. Figure 2 clearly shows that the fusion weights maintained stable when the cluster number, which hardly influenced the contributions of omics to clustering objects, varied. Also, when the protein DE or RNA DE varied, the fusion weights changed adaptively and are positively correlated with the quality of the omics data.

### 3.2 Real data experiments

#### 3.2.1 scCTClust is powerful clustering CITE-seq data

**FIGURE 3:**
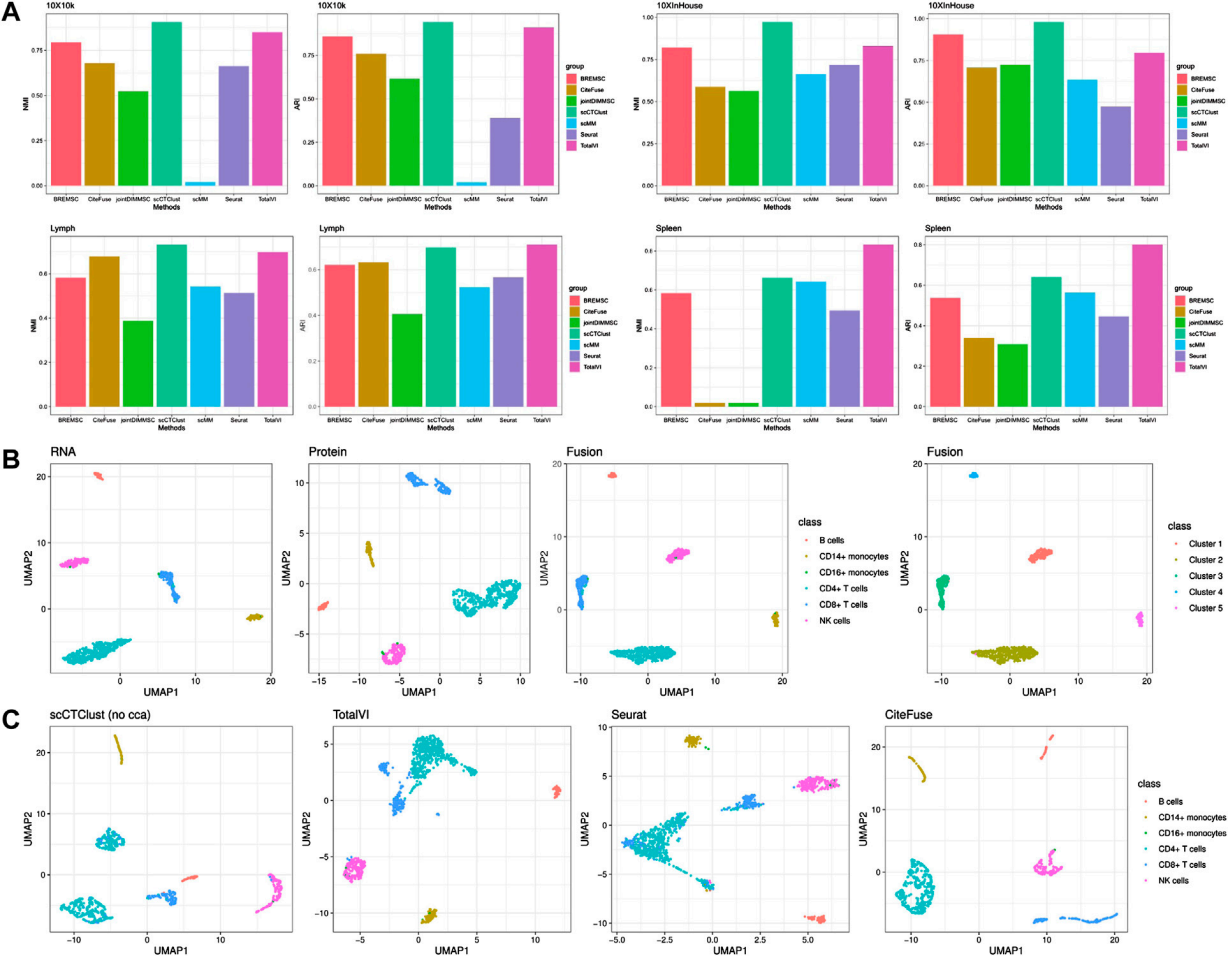
CITE-seq Experiments. **(A)** Performance of scCTClust and competing methods by NMI and ARI over real CITE-seq data sets 10X10 k, 10XInhouse, Lymph, and Spleen. **(B)** UMAP visualization of RNA, protein, and fused features applying scCTClust over the 10XInHouse data set; the left three are colored by true cell types, and the right one is colored by the predictions. **(C)** UMAP visualization of latent features extracted by competing integrative methods, namely, scCTClust trained without CCA loss, TotalVI, Seurat, and CiteFuse, over the 10XInHouse data set.

To further confirm the effectiveness of scCTClust, we selected four real-world CITE-seq data sets on which scCTClust and competing methods were applied. In specific, the competing methods include Seurat V4, scMM (Minoura et al., 2021), CiteFuse, joint DIMM-SC (Sun et al., 2018), BREM-SC, and TotalVI. First, we evaluated the performance of scCTClust and competing methods applied to all four data sets by NMI and ARI. As is shown in Figure 3, scCTClust is evidently superior to other methods on data sets 10X10 k and 10XInhouse, achieves similar NMI and ARI against TotalVI on the Spleen data set, and is slightly worse than TotalVI on the Spleen data set. TotalVI gained better NMI and ARI than Seurat and CiteFuse, indicating the advantage of a deep learning algorithm in characterizing the features of high-dimensional data. BREM-SC behaved pretty well in these two data sets, especially over the 10XInHouse data set, with its ARI approaching our scCTClust. However, utilizing a vanilla MCMC, BREM-SC can hardly be praised as scalable, taking hours over the 10X10 k data set with 7865 cells. Second, we used the two-dimensional visualization method UMAP to investigate the latent structure of scCTClust. We showed fused representation and RNA and protein low-dimensional representations of scCTClust applied to the 10XInHouse data set, colored with the true cell types. We can see that although the protein representation was not well suited for clustering, scCTClust managed to fuse it with RNA representation and obtain a fusion representation with compact and separable clusters and thus obtaine accurate predictions. Third, we plotted the UMAP visualization of latent features extracted by TotalVI, Seurat, CiteFuse, and scCTClust trained without optimizing CCA loss. We can see that the clusters of these three competing methods are not only less compact but also less separable than scCTClust. Additionally, CD8^+^ cells were assigned to two clusters by Seurat. Casting out sight to the UMAP plot of scCTClust (no cca), CD4^+^ cells have also been divided into two clusters. This phenomenon indicates that the representations of two omics have not been properly combined. We also colored the UMAP plot of fusion representation by the expression of surface proteins in Supplementary Figure S2. For instance, we can clearly see that protein CD56 is highly expressed in NK cells. It demonstrated that scCTClust can help us verify and may discover new correlations between the expressions of surface proteins and cell types.

#### 3.2.2 scCTClust can also cluster SNARE-seq data

Although scCTClust is designed for CITE-seq data clustering analysis, it is well modularized and can easily be generalized to cluster other multiomics data. We selected applied scCTClust and several state-of-the-art multiomics clustering methods on two transcriptome–epigenome data sets. The two data sets contain a SNARE-seq (Jin et al., 2020) CellLine data set and a SHARE-seq (Ma et al., 2020) Ma data set. The competing methods are, namely, Seurat V4, MOFA+, scAI, scMVAE (Zuo and Chen, 2021), and DCCA (Zuo et al., 2021). The performance of each method was evaluated by evaluation index NMI and ARI.

To adapt scCTClust on SNARE-seq data, we converted the peak level count matrix of scATAC-seq data to the gene activity data like gene expression values of the scRNA-seq data and modeled each omics data drawn from one zero-inflated negative binomial (ZINB) distribution. In this way, the large dimensional difference between scRNA-seq and scATAC-seq data can be balanced. The training loss for scCTClust still follows Eq. 17, while the ZINB loss consists of two parts, ZINB loss for RNA and ZINB loss for ATAC.

**FIGURE 4:**
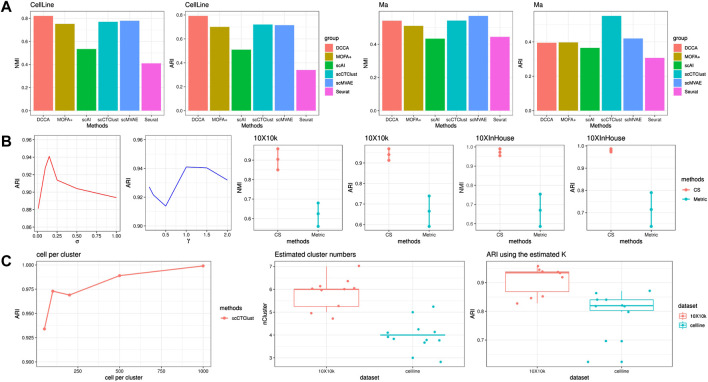
SNARE-seq experiments and ablation studies. **(A)** Performance of scCTClust and competing methods by NMI and ARI over the SNARE-seq data set CellLine and SHARE-seq data set Ma. **(B)** Ablation study to determine the robustness of hyperparameters and the advantages of C-S divergence-based clustering against metric-based clustering. **(C)** Performance of scCTClust over different scales of simulated data sets. The estimated number of clusters and clustering results were obtained with it.

We displayed the NMI and ARI of scCTClust and competing methods applied to CellLine and Ma data sets in Figure 4. On the four-cluster CellLine data sets, scCTClust gained similar performance comparing scMVAE and is slightly poorer than DCCA. However, tuning the hyperparameters of DCCA, especially tuning the loops for cross-omics cycle attention, is quite time-consuming. Matrix factorization-based methods MOFA+ and scAI did not present a significant performance, ranking in the fourth and fifth positions of the six methods in NMI and ARI. Seurat performed poorly with an NMI of around 30%. Ma is a large-scale mouse skin data set with 34,774 cells. When applied on Ma-2020, scCTClust obtained the best ARI over six methods and got an NMI ranking second. DCCA gained an NMI and an ARI lower than scCTClust and scMVAE. Although the attention mechanism adopted by DCCA has potential in integrative analysis, the low-quality part of highly sparse ATAC data easily perturbs RNA feature extracting through network feedback. Additionally, as the number of different cell types concluded in Ma comes to a number of 22, it is especially hard for the highly sparse and less informative, ATAC-seq data to accurately distinguish all cell types. As illustrated in the introduction, the power of this kind of alignment rapidly deteriorates, which may account for the unsatisfactory performance of DCCA. scAI and MOFA again presented ordinary results, ranking fourth and fifth of all six methods. In addition, matrix factorization-based methods were far less scalable than deep learning methods, spending days on large data sets such as Ma. Overall, the performance of scCTClust well demonstrated that it has great potential for generalizing to cluster other single-cell multiomics data.

### 3.3 Ablation studies

#### 3.3.1 Canonical correlation analysis finds shared space suited for clustering

In Figure 3, we plotted the two-dimensional visualization of fusion representations learned by scCTClust, trained with or without CCA loss, over the 10XInHouse data set. We can clearly see that without improving the correlation between two omics representations, scCTClust trained without $L_{\mathrm{cca}}$ divided CD4^+^ T cells into two clusters and got less compact clusters for NK cells and CD14^+^ monocytes, compared to scCTClust trained with $L_{\mathrm{cca}}$ . It illustrated that improving the correlation helps find a better space for clustering. Comparing the visualization of fusion representations to RNA and protein representations extracted by scCTClust, we can see that after fusion, the clusters became more compact and separable than any single omics representation. It demonstrates that CCA loss helps effectively integrate multiomics representations.

#### 3.3.2 C-S divergence-based clustering is more robust than metrics-based clustering

To demonstrate the advantages of C-S divergence-based clustering against the conventional metric-based clustering, we replaced the present clustering module in scCTClust with a deep embedded clustering one (Xie et al., 2016). We applied these two implements of scCTClust on 10X10 k and 10XInHouse; each experiment is repeated with 10 different random seeds. The mean NMI ± the variance of NMI is depicted in Figure 4, and we can see that our C-S divergence-based clustering got a higher mean and lower variance, indicating that it is more accurate and more robust than metric-based clustering.

#### 3.3.3 scCTClust is not sensitive to hyperparameters

We also verified stability, while varying two hyperparameters introduced in our model, including *σ* and *γ*, which can be seen in Figure 4. We fix the training data set and CITE-seq data set at 10X10 k and tuned those hyperparameters to observe any change in scCTClust’s performance. We found the performance of scCTClust to be insensitive to *γ*. We also found that the adequate intervals for *σ* were similar across different experiments.

#### 3.3.4 scCTClust can cluster small-scale data

One of the limitations of many machine learning models (especially neural networks) is the requirement of a large number of input data, to allow for a robust estimation of the model parameters and hyperparameters. To investigate whether scCTClust can still perform well when data are relatively insufficient, we applied scCTClust over a series of simulated CITE-seq data, generated by the R package Splatter. We only vary the number of cells in each cluster as (50, 100, 200, 500, and 1000), with each simulated data set keeping (n_cluster, n_protein, DE_RNA, DE_protein) = (8, 75, 0.15, 0.7). The performance of scCTClust is evaluated with ARI and is shown in the form of a line chart in Figure 4. We can see that the performance of scCTClust slightly decreased as the size of the input data set became smaller. The clustering result for the data set with the smallest scale is still trustworthy.

#### 3.3.5 The selection of cluster number *K*

Through all experiments, including scCTClust and competing methods ones, we set the parameter *K* referring to the number of clusters as the true different cell types contained in the according data set. In practice, when the number of different cell types is not attainable, we suggest determining the value of *K* based on the k-means algorithm and evaluate the index SSE (sum of squared error).

To be specific, we first pre-train the scCTClust model merely with the loss for omics-specific autoencoders $L_{AE}$ . By doing this, we obtain a fused latent representation *z* on which we apply the k-means algorithm with different values of *K* to obtain a clustering result. We compute SSEs with those clustering results and plot the SSE-K line chart. The best value of *K* is determined as the inflection point of the chart. We repeated the aforementioned methods 10 times over the 10X10 k and CellLine data set and trained scCTClust with the learned *K* each time. The evaluated *K* and the ARI of the clustering result using that estimate are shown in Figure 4. We can see that scCTClust estimates the number of clusters accurately and can still perform well when the parameter referring to the number of clusters is slightly biased.

## 4 Conclusion and discussion

Clustering CITE-seq data is a challenging job. CITE-seq data inherit the large dimension and high sparsity from scRNA-seq data. Clustering CITE-seq data encounter new obstacles such as the dimension difference between omics, the difficulty to quantify the contributions of omics to the clustering object, and the probable low correlation between the representations of omics. To address these problems, we proposed scCTClust. Our scCTClust is equipped with omics-specific encoders to extract omics features separately. scCTClust utilizes ZINB and NB models to characterize two different omics data sets and introduces a CCA loss to maximize the correlation between omics representations. A decentralized clustering module is set after the linear combination of omics representations. The combining weights are adaptively chosen during training to quantify the contribution of two omics to clustering objects. Extensive real-world and simulated CITE-seq experiments have illustrated the effectiveness and efficiency of scCTClust. SNARE-seq and SHARE-seq experiments revealed that scCTClust can be easily generalized to cluster other multiomics data.

However, we also find several shortages of scCTClust in the experiments. First, scCTClust does singular value decomposition (SVD) when computing the correlation between latent representations of omics data and thus encounter a problem of numerical instability. Stopping the use of all components and use of the top components instead can alleviate this problem in most cases. Second, in Figure 2, when clustering the six-cluster 10XInHouse data set, although scCTClust achieved high NMI and ARI, scCTClust failed to distinguish the rare cell type ‘CD16+ monocytes’ from NK cells. Since there are only seven samples from ‘CD16+ monocytes’ out of a total of 1182 samples, identifying them is a tough job indeed. However, we will still make our best efforts to address these two problems.

## Data Availability

Publicly available data sets were analyzed in this study. This data can be found here: https://github.com/ddb-qiwang/scCTClust-torch.
