# Supplementary figures and images for: Clustering CITE-seq data with a canonical correlation-based deep learning method
Source: Front Genet. 2022 Aug 22;13:977968. doi: 10.3389/fgene.2022.977968 (PMC9441595; doi:10.3389/fgene.2022.977968)

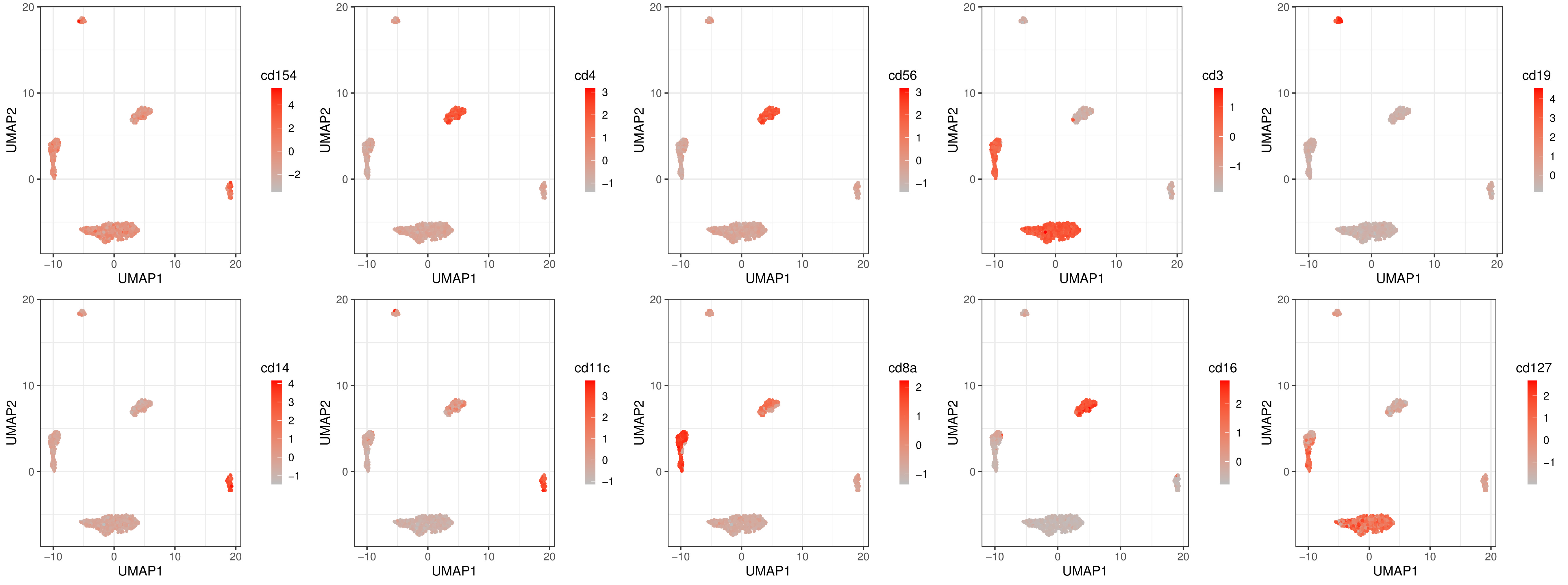

Supplement: Supplementary file 1 [file Image2.TIF]

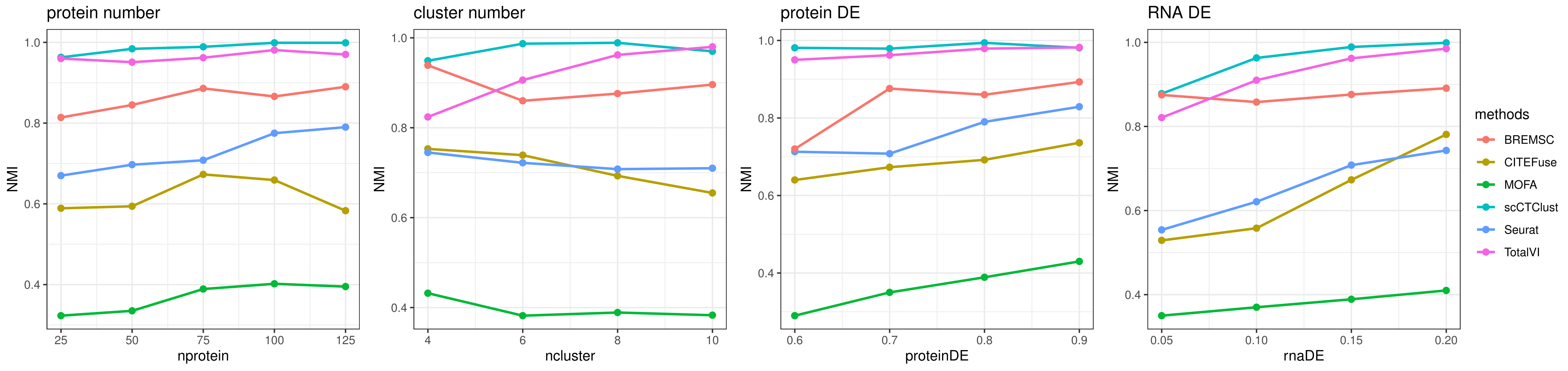

Supplement: Supplementary file 2 [file Image1.TIF]
